# The Influence of Emotional Intelligence on Performance in Competitive Sports: A Meta-Analytical Investigation

**DOI:** 10.3390/sports6040175

**Published:** 2018-12-13

**Authors:** Alexandra Kopp, Darko Jekauc

**Affiliations:** 1 Department of Sport Science, Humboldt-Universität zu Berlin, 10115 Berlin, Germany; 2 Department of Sport Science, Karlsruhe Institut of Technology, 76131 Karlsruhe, Germany; darkojekauc@kit.edu

**Keywords:** emotional intelligence, emotional competence, sports performance, competitive sports, meta-analysis

## Abstract

Emotional intelligence (EI) is considered as a factor influencing sport performance. The research findings are inconsistent with respect to the size and even the direction of the relationship, however. In order to summarise the available evidence, we conducted a meta-analysis examining the relationship between emotional intelligence (EI) and sports performance in competitive sports. A systematic literature search was conducted in June 2018. We identified 21 studies targeting EI and sports performance in competitive sports. We calculated correlation (r) to estimate the effect of the relationship. A random effects model was used to interpret findings. The meta-analysis of 22 effect sizes on the response of 3.431 participants found a small but significant relationship between EI and sports performance (*r* = 0.16). Additionally, the conceptualisation of EI (ability concept, trait concept, or mixed-model concept), type of publication, citation counts, and publication date turned out not to be significant moderators. Overall, the result is encouraging regarding the value of EI as a possible predictor in sports performance.

## 1. Introduction

Emotion is an inherent part of the competitive experience [1,2,3]. According to Kleinginna et al. [4] emotion can be understood as a complex set of interactions among subjective and objective factors, mediated by neural-hormonal systems, which can (a) give rise to affective experiences such as feelings of arousal, pleasure/displeasure; (b) generate cognitive processes such as emotionally relevant perceptual effects, appraisals, labeling processes; (c) activate widespread physiological adjustments to the arousing conditions; and (d) lead to behavior that is often, but not always, expressive, goal-directed, and adaptive.

There is a consensus that emotions have an important impact on athletic performance [1,5,6,7]. Emotions influence perception, cognition, neurophysiology, motivation, behavior, motor expression, subject feeling and decisions, thereby either facilitating or debilitating sport performance [1,3,8,9]. Meta-analytic studies have highlighted the link between emotions and performance [9,10]. For example, Beedie et al. [9] found small to moderate effects associated with level of performance. Craft et al. [10] evaluated the anxiety and performance relationships in competitive sport and their results demonstrated that athlete’s performance is successful when his or her pre-competition anxiety is within or near the individually optimal zone. With that, athletes’ ability to identify and understand emotions, and to regulate them in order to achieve optimal performance was brought into the focus of sport psychology [8]. Reflection of emotion is analogous to the construct of emotional intelligence (EI) [11].

EI is defined as ‘‘the ability to perceive and express emotion, assimilate emotion in thought, understand and reason with emotion, and regulate emotion in the self and others’’ [12]. In the EI tradition, individual differences are studied with regard to how subjects identify, understand, express, regulate, and use their own emotions and those of others [11]. The concept of EI, introduced in the scientific literature in 1990 by Mayer and Salovey, and undoubtedly made popular by Daniel Goleman [13], has generated broad interest within the field of psychology [14,15,16]. This interest in the topic was initially fueled by anecdotal evidence suggesting that mental ability by itself is not enough for success in life [17]. The EI area has grown exponentially over the past three decades in various disciplines [18]. As should also be mentioned, however, the construct was met with a sizeable critical response with regard to the concept, theory, and measurement of EI from the beginning [15,18,19,20]. A large body of studies highlighted the importance of EI as a predictor in important domains such as academic performance, job performance, leadership, stress, better health, and well-being [18]; meta-analytic results systematised the links between EI and these important life success variables [21,22,23,24,25,26]. For example, Perera et al. [24] found a moderate positive relationship of trait EI and academic performance. O’Boyle et al.’s [27] and Joseph et al.’s [28] results demonstrated that EI is positively associated with job performance. Schutte et al. [21] and Martins et al. [22] demonstrated a positive correlation between EI and health. Finally, the meta-analytic results of Sánchez-Álvarez et al. [25] demonstrated that EI is positively associated with well-being. Consequently, it is not surprising that the interest of applied sport psychology in the concept of EI has also grown substantially. Martinez et al. [29] reported their review that initial research into EI in sports stems from 2001 [29]. As mentioned earlier, interest in examining the nature of the role of EI in sports settings has grown constantly, which is reflected in the number of studies in a variety of settings in sports [5,29,30,31].

### 1.1. Emotional Intelligence and Sports Performance

There is no doubt that competitive sport is an emotion-laden environment. First, athletes are continuously faced with various types of stressors and emotional challenges inside and outside competition, linked to their athletic performance [6]. Second, athletic performance is a result of relationships and interactions and these are significantly influenced by emotions [32,33]. Athletes have to be aware of the feelings of other members (e.g., teammates, coaching staff, opponents, officials, family, fans and sport administrators). They have to communicate and work together to reach their best performance [34]. In this respect, athletes need meta-emotional beliefs in how their emotions influence their performance [6]. They must learn to recognise their own emotions and ideal performance states to control their level of energy in order to achieve optimal performance [1,35,36]. Some studies have investigated the effects of EI on team performance and individual performance parameters in specific sports (e.g., cricket, baseball, hockey, basketball, tennis, ballet, etc.), and their findings vary [37,38,39,40]. For example, Crombie et al. [37] found that the average team ability EI was positively related to the team performance of cricketers. Also, Petrides et al. [40] reported a positive correlation between trait EI and ballet dancing ability ratings. Zizzi et al. [34] found that components of EI appear to be moderately related to pitching performance, but unrelated to hitting performance in baseball. Also, Laborde et al. [38] found that trait-EI was unrelated to tennis serve performance. Similarly, Perlini et al. [39] found no significant relation between performance measurements and EI in ice hockey athletes. In their systematic review of EI in sport and physical activity, Laborde et al. [5] summarised the results of six studies investigating the relation of EI on athletic performance [34,38,39,41]. They concluded that in the context of sports performance, EI relates to emotions, physiological stress responses, successful psychological skill usage, and more successful athletic performance [5]. They also reported contradictory findings [42] and a need for further scientific work to explore the connection between EI and sports performance in more detail.

Taken together, the findings provide some initial evidence that EI has a connection to sports performance. However, the magnitude and direction of the relationship between EI and sports performance varies considerably. It should be noted that a discrete report from a series of studies does not constitute a valid mechanism for synthesising data. Rather, in working with a collection of studies, one needs to answer the question whether or not the effect size is consistent across studies. A meta-analysis provides a mathematically rigorous mechanism for this purpose [43]. In the current investigation, meta-analytic methods were used to quantitatively summarise empirical research assessing the effect of the relation between EI and sports performance. An overall effect size was obtained to answer the following research question:

Research Question 1: Is EI related to sports performance in competitive sports?

### 1.2. Potential Moderators for the Relationship of Emotional Intelligence (EI) and Sports Performance

In addition to the main effect, we were interested in several potential conceptual and methodical moderators of the relationship between EI and sports performance in competitive sports. We introduce these moderators below.

#### 1.2.1. Type of Sport as a Potential Moderator

Laborde, Guillén, and Mosley [44] stated that the type of sports practiced is an important distinction. Such a distinction is often conceptualised as either individual or team sport [44]. Furthermore, Laborde, Guillén, et al. [44] showed examples for the different psychological requirements. In individual sports, the actions and decisions cannot be compensated for by teammates. This implies that an individual athlete has more of the responsibility for a competitive outcome. Thus, individual personality characteristics play a major role in determining the result. In addition, EI research demonstrated the difference in personality characteristics between individual athletes and team sport athletes [45,46]. It should, therefore, be interesting to explore whether effect sizes vary depending on the type of sport. The moderator analysis of the type of sport on the effect of the relationship between EI and sports performance will be exploratory in nature. Thus, we examined the following research question:

Research Question 2: Is there a difference between effect size between individual sports athletes and team sports athletes?

#### 1.2.2. Level of Expertise in Sports as a Potential Moderator

Sport psychology research is interested in a better understanding of the psychological factors distinguishing outstanding individuals from less outstanding ones in sport [47]. In this context, the answer to the question of whether successful competitive athletes differ in their personality structure from less successful ones is one main issue [48]. EI research in sports has, however, speculated that EI differs across sport expertise [6,49,50]. Laborde et al. [44] reported that mean EI scores did not differentiate between athletes with different levels of expertise. Vaughan et al. [51] examined the differences in EI across elite, amateur, and non-athletes and concluded that EI does not differ across sport expertise. However, there are too few comparative studies directly examining differences across sports expertise to draw any meaningful conclusion. Hence it will be interesting to explore whether or not effect sizes vary depending on the level of expertise. The moderator analysis of level of expertise in sports on the effect of the relationship between EI and sports performance will be exploratory in nature. Consequently, we examined the following research question:

Research Question 3: Does effect size depend on sports expertise?

#### 1.2.3. Measurement of Sports Performance as a Potential Moderator

A performance indicator is a selection, or combination, of action variables intended to define some or all aspects of a specific performance [52]. Sports performance can be operationalised in quite different ways. There are, for example, objective measures (points or times), league membership, subjective ratings by coaches or self-assessment, and categories reflecting performance differences such as “qualified versus not qualified for the Olympic team” or “win versus loss” [53]. Previous studies on the relationship between EI and sports performance are characterised by a heterogeneous use of different sports performance measurements [37,38,40,41]. It should then be interesting to explore whether effect sizes vary depending on the type of measurement of sports performance. The moderator analysis of measurement of sports performance on the effect of the relationship between EI and sports performance will be exploratory in nature. Thus, we explored following research question:

Research Question 4: Does effect size depend on measurements of sports performance?

#### 1.2.4. Conceptualisation and Measurement of EI as Potential Moderator

Within the theoretical approach to EI two major distinct perspectives are discussed: the ability concept and the trait concept of EI, which vary widely in both content and method of assessment [15,16,17,54]

The ability approach conceptualises EI as a cognitive ability based on the processing of emotion information [55] and comprises a set of abilities that can be learned and developed over time. The mainstream model of ability EI is the four branch model introduced by Mayer and Salovey [11], distinguishing between four areas of problem-solving necessary to carry out emotional reasoning: (a) perceiving emotions, (b) facilitating thought by using emotions, (c) understanding emotions, and (d) managing emotions in oneself and others [56]. Ability EI is assessed with maximum-performance tests [11] requiring individuals to demonstrate their ability EI in response to a variety of hypothetical scenarios [55]. The most used ability-measurement is the Mayer–Salovey–Caruso emotional intelligence test (MSCEIT) [17].

The trait theory was introduced by Petrides [57] and the trait model can be understood as a cluster of lower order personality dispositions capturing the extent to reflect the way people usually deal with their own and others’ emotions [58]. The model has four components: well-being, sociability, self-control, and emotionality [17,59]. Trait EI is typically assessed via self-report questionnaires [57], for instance with the Trait Emotional Intelligence Questionnaire (TEIQue) [57,59].

Alongside these two approaches, there are so-called mixed models of EI including social skills, motivation, self-esteem, and personality aspects. The main components of these models are intrapersonal skills, interpersonal skills, adaptability, stress management, and general mood [60] (Bar-On, 1997, 2006). Mixed models are also operationalised via self-report measurements [60], such as for instance the emotional quotient inventory (EQ-i), a self-report measure developed by Bar-On [17].

Previous applied sport psychology research is characterised by a heterogeneous use of different conceptualisations and measurements of EI [5,6]. This raises the question of whether effect sizes vary depending on the type of EI concepts and the type of EI measurement. The moderator analysis of EI concepts and EI measurement on the effect of the relationship between EI and sports performance will be exploratory in nature. Accordingly, we explored following research questions:

Research Question 5: Does effect size depend on the conceptualisation of EI?

Research Question 6: Is there a difference in effect size between assessment of EI via self-report and assessment of EI via ability-test?

#### 1.2.5. Quality Criteria as Potential Moderators

Evaluation based on scientific publications involves, for example, the analysis of citation counts or the analysis of peer-reviewed status. While the former reflects the evaluation of the attention paid to the publication, the latter is an assessment of content. Regarding these methods for evaluating publications, we have no theoretical assumptions to expect an effect on the relationship between EI and sports performance. However, it will be interesting to explore the question of whether effect sizes vary. The same also applies to the date of publication. The moderator analysis of citation counts, peer-reviewed status and published date on the effect of the relationship between EI and sports performance will be exploratory in nature.

Research Question 7: Does effect size depend on quality criteria?

In sum, the aim of our study was to provide a meta-analysis on the relationship between EI and sports performance in competitive sports. No prior meta-analyses have examined EI and sports performance, so this relationship has not been tested using meta-analytical techniques. We accurately determined the overall size of the relationship and, in addition to this main effect, we examined several potential conceptual and empirical moderators of the relationship between EI and sports performance.

## 2. Methods

### 2.1. Procedure

#### 2.1.1. Exclusion and Inclusion Criteria

To be included, studies had to meet the following criteria: (a) the study was empirical in nature; (b) the study involved quantitative methods; (c) the study had assessed EI and sports performance; (d) the sample was constituted of sport competition participants; (e) the study presented a quantitative correlation analysis or comparison design; and (f) the article was written in English, German, or Spanish. In those cases where a study used multiple samples, each sample could be included as a separate entry, as long as the study met the inclusion criteria. We included dissertations and unpublished reports in addition to studies found in scientific journals. Two reviewers independently screened titles with respect to the inclusion criteria. After title screening, articles were subjected to an abstract screening and checked for duplications. After excluding duplicated records, articles were subjected to a full text screening. Any discrepancies were resolved by discussion with a third reviewer. 

#### 2.1.2. Literature Search

The literature search intended to identify relevant published studies that have explored EI with a specific focus on sports performance in competitive sports. We adhered to the PRISMA guidelines (Preferred Reporting Items for Systematic Reviews and Meta-Analyses) [61]; a copy of the PRISMA checklist and a protocol of the meta-analysis (Table S1) are available from the authors on request. The search was conducted on 28 June 2018, and included the following keywords: “emotional intelligence,” or “emotional competences,” and “sport,” or “athletes,” or “competitive sport,” or “sports performance”. Search engines used included SPORTDiscus, PsycARTICLES, PsycINFO, Scopus, and Web of Science. We did not limit research period. We also searched for all studies referenced in papers identified in this search.

### 2.2. Data Preparation

#### 2.2.1. Extraction of Data

Initially, the first author extracted data with to the following key features: reference information, aim of the study, type of study design, participants and sample characteristics, type of sport, EI theory or rather conceptualisation, dependent variables and how they were measured, instrument used for measuring EI and their psychometric characteristic, data analysis, statistical outcomes and, if available, effect size.

#### 2.2.2. Extraction of Effect Sizes

Correlation (*r*) was used as the effect size because the majority of the research reported *r*, as it is easily calculated from chi-square, *t*, *f*, and *d* values, and it is readily understood and interpreted [62]. For studies based on group differences that did not report the effect size, but had given enough details to calculate an effect size, we first calculated the standardised mean differences (Cohen’s *d*) and computed this effect size to *r* [63]. If the same EI measurement was correlated with multiple performance measurements, provided they belonged to the same type of performance measurement, we averaged the correlations to obtain the effect size [64].

#### 2.2.3. Coding of Studies

After a first data extraction, studies were coded for the data analysis. To this end we coded the conceptualisation of EI according to the typical classification in literature [65]: (a) ability approach to emotional intelligence; (b) mixed models of emotional intelligence; (c) trait approach to emotional intelligence. For the type of EI measurement we distinguished between self-report and ability-test. In an effort to integrate research findings despite different instruments used for measuring EI and their psychometric characteristic, we used the global score of EI where available. If the total score of EI was not reported, but the results of all EI subscales were available, an average correlation was calculated from the scales to obtain the effect size, a practice consistent with the guidelines provided by Hunter and Schmidt [64]. We coded the type of sport into three categories (individual-sport, team-sport, or both together). We identified four categories used at level of expertise in sports (elite athletes, professional athletes, amateur athletes, and studies which included all of them). For the type of sports performance measurement we also identified four different categories (a) level of expertise; (b) statistical accounts; (c) assessment of sports performance; and (d) physiological parameters. For the year of publication we divided the studies into two homogeneous groups which could provide a basis for comparison (studies published between 2001 and 2009, and studies published since 2010). Furthermore, we made a distinction between (a) peer-reviewed documents and (b) non peer-reviewed documents. Finally, the number of citations in Google Scholar was counted on 3rd August 2018 and we sorted into three different categories for the number of citations (0–10, 11–50, and >50). All studies were coded independently by the authors, and their ratings were compared. Any disagreements were resolved by discussion and consensus.

#### 2.2.4. Coding of Quality

Quality evaluation was carried out according to the guideline of the Strengthening the Reporting of Observational Studies in Epidemiology (STROBE) statement to assess the quality of the studies included in this meta-analysis [66]. The STROBE statement contributes towards improving the quality of reporting of observational studies by using a checklist of 22 items. In the present meta-analysis, 15 items of the STROBE-statement were deemed necessary for the purpose of evaluating the quality of the studies chosen by the authors. We rated the items as (a) available, (b) partly available or (c) not available. Any disagreement was resolved by discussion. If consensus was not possible, a third researcher resolved the issue. Additionally, we counted the number of citations in Google Scholar and checked whether the journal was peer-reviewed.

### 2.3. Statistical Analysis

#### 2.3.1. Meta-Analytic Procedure and Assessment of Publication Bias

Comprehensive Meta-Analysis software (CMA), Version 2.0, (Biostat, Inc., Englewood, NJ, USA) was used. A random effects model was used to interpret findings [64,67]. A random effects model assumes that error is connected to sampling procedures and additional between-study variance [68,69]. Analyses completed using a random effects model adjusts effect sizes by the inverse weight of the variance in order to consider both the sampling and between-study error [43]. Interpretation of the effect size calculations were based on Cohen’s [70] determination of small (*r* ≤ 0.10), medium (*r* ≤ 0.30), and large (*r* ≥ 0.50) effect sizes.

The *I*-square (*I*^2^) value provides an estimate of the overlap of confidence intervals and is interpreted as low (25%), moderate (50%), or high (75%) values of the total variance attributed to covariates [71]. If *I*^2^ is high, then heterogeneity is very high, and the use of the random effect model for meta-analysis is justified [72]. Decisions made concerning retention or exclusion of outliers, i.e., large residual values two standard deviations (±1.96) above or below the study’s mean effect size, were based on whether overall results remained significant (*p* ≤ 0.05) and within the 95% confidence interval. The influence of individual studies on the overall mean weighted *r* was investigated by removing one study at a time from the overall analysis (one-study removed analysis) by using a ‘one study removed’ procedure in CMA.

Meta-analytic results can also be influenced when relevant studies are missed or excluded [73,74]. Methods used to identify and control for publication bias included review of the funnel plot, the Egger’s test for detecting asymmetry in a funnel plot [75], a Fail-safe N calculation [73], and a ‘trim and fill’ procedure [76]. Each study included the number of participants, the effect size (Pearson’s *r*), the confidence interval (lower limit, upper limit), the relative weight, the residual value and the summary effect size if the study removed it.

#### 2.3.2. Moderator Analysis

Moderator analysis provides the strength and/or direction of relationships between independent and dependent variables [43]. We used *Q* statistic to test differences across moderators. A statistically significant *Q* statistic suggests that there is heterogeneity in effect size distribution (i.e., the potential existence of moderators). We only examined moderators when the number of studies in the subgroups was large enough to justify doing so.

## 3. Results

### 3.1. Sample and Study Characteristics

#### 3.1.1. Sample

**Figure 1:**
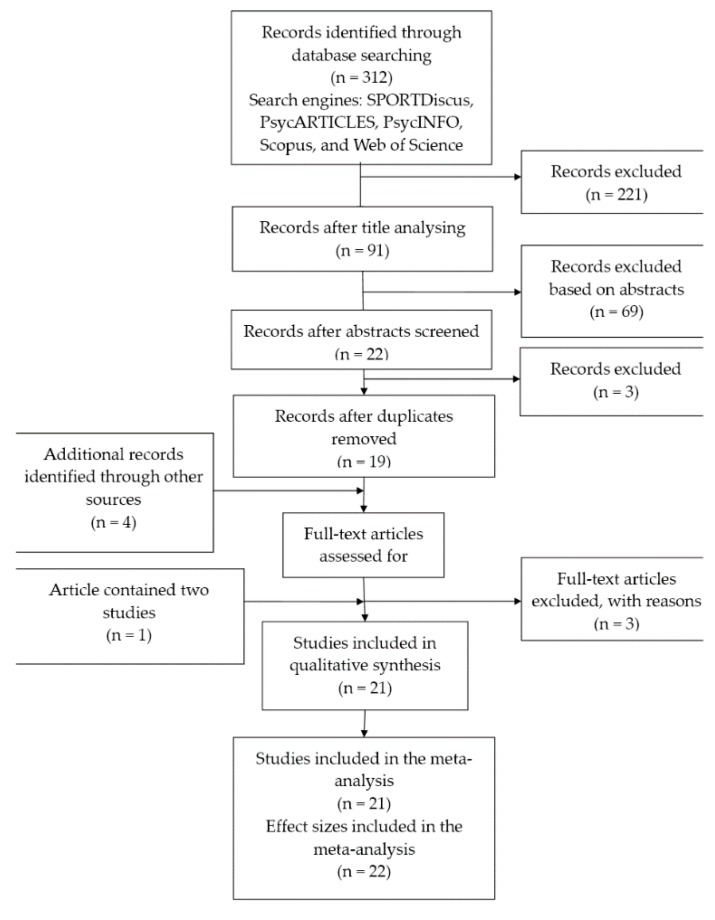
Flow diagram of the review process.

The search identified 312 results. Ninety one articles were subjected to an abstract screening. Nineteen articles were subjected to a full text screening. Also, four additional records were identified via the reference lists. Three articles were excluded because of non-compliance with criterion (c), one article contained two studies [38]. Finally, 21 articles targeting EI and sports performance in competitive sports were retained for the meta-analysis. The 21 studies identified were published between 2001 and 2018, with most studies (33.3%; *k* = 7) published in 2014. In total, 17 studies were scientific journal articles, 12 of them were peer-reviewed. Three studies were dissertations and one article was published in a book. Figure 1 provides a detailed overview of this search.

The overall sample size was *N* = 3.431. The sample consisted of 57.1% analyses that reported a higher percentage of males than females in their samples. In four studies no information about gender is provided. The age in the respective studies demonstrated a big range (min = 16.0, max = 56.0).

#### 3.1.2. Type of Sport

The studies were heterogeneous in terms of type of sport. Forty-eight percent (*k* = 10) of the analyses were carried out on data collected from team-sport athletes, 23.8% (*k* = 5) from individual-sport athletes and 19.1% (*k* = 4) refer to both types of sport. For two studies it was not possible to make an allocation.

#### 3.1.3. Level of Expertise in Sports

The analyses included were also heterogeneous in terms of the participants’ performance level. Most analyses were performed on data collected from professional athletes (52.4%; *k* = 11) or a mixed sample (23.8%; *k* = 5), ranging from elite to amateur athletes. Two studies were conducted using data solely consisting of elite athletes, and one study was carried out with data collected from amateur athletes. In two studies no information about performance level was provided. We were not able to give precise statistical information on social and demographic factors because this data was not reported with sufficient clarity across the studies.

#### 3.1.4. Measurement of Sports Performance

Sports performance was measured most frequently via statistical accounts (47.6%; *k* = 10). Level of expertise respectively league-membership were used in six studies (28.6%). Four studies used an assessment of sports performance such as evaluation by coaches or athletes themselves. One study used physical parameters, namely the maximum voluntary contraction (MVC) [41].

#### 3.1.5. Conceptualisation and Measurement of EI

The analysis of the conceptualisation of EI demonstrated that 23.8% (*k* = 5) of the studies assigned themselves to the mixed models of emotional intelligence, respectively, to the trait approach to emotional intelligence. Four studies assigned themselves to the ability approach to emotional intelligence. In seven studies no details about the type of EI concept was provided.

A very strong heterogeneity can be seen above all in the measuring instruments used in the analyses. In 21 studies, 14 different measurements of EI were applied, while the Trait Emotional Intelligence Questionnaire (TEIQue, [59]) was the measurement most used (*k* = 4).

With regard to the type of EI measurement, a fairly uniform picture is apparent. In almost all studies EI was measured via a self- report test (90.5%; *k* = 19), whereas only two studies used an ability-test for measuring EI [37,77]. Analyses focused mainly on the overall score of EI and its subscale scores, while six studies only reported an overall score of EI and four studies were limited to the report of subscale scores. A summary of the studies is provided in Table 1.

#### 3.1.6. Quality Criteria

Analyses were mainly cross-sectional studies (90.3%; *k* = 19). One study was performed on data from a quasi-experimental design [42] and one study was characterised by a longitudinal research design [37]. Fifteen studies can be described as peer-reviewed studies. The number of citations ranged from 0 to 159.

The quality evaluation by using selected items of the STROBE-statement showed that the majority of the analyses reported sources of data and details of methods of assessment for each variable of interest in their study (85.7%; *k* = 18). Eighty one percent (*k* = 17) of the studies stated specific objectives and reported numbers of outcome events or summary measurements. More than half of the studies described all statistical methods (61%; *k* = 13). Finally, 52% (*k* = 11) of the analyses stated pre-specified hypotheses and presented key elements of the study design early in the paper. In contrast, one study reported power calculations, almost none of the analyses (95.2%; *k* = 20) explained how the study size was arrived at, they did not describe any efforts to address potential sources of bias, and they did not explain how missing data were dealt with. Furthermore, more than half of the studies did not provide the methods of selection of participants, and did not explain how quantitative variables were handled in the analyses (57%; *k* = 12). Finally, 11 studies did not discuss limitations of the study and did not discuss the generalisability of the study results. A detailed overview is provided in Table 2.

### 3.2. Summary of Statistical Analysis

#### 3.2.1. Meta-Analytic Effect and Publication Bias

The studies available for the meta-analysis on the relationship between EI and sports performance included a total of 22 effect sizes of independent samples (*N* = 3431). The results showed a significant positive correlation (*k* = 22, *r* = 0.16, 95% confidence interval, *CI* [0.11, 0.22], *Z* = 5.44, *p* = 0.00). This can be considered as a small effect [70]. The Q statistic and the *I*^2^ index indicated heterogeneity of the sample (*Q* = 43.40; *I*^2^ = 51.61). The overall mean weighted r was not influenced by any one study. Two studies had a large residual value [37,39]. The total effect excluding these two studies was still significant, also demonstrating a small effect (*k* = 20, *r* = 0.17, 95% confidence interval, *CI* [0.12, 0.22], *Z* = 6.33, *p* = 0.00).

The funnel plot showed that the effect sizes in the individual studies were symmetrical at the top and relatively symmetrical in the middle. Near the bottom, studies were missed and the few remaining studies were located on the right side of the overall mean weighted effect size. This may indicate the presence of publication bias (A copy of the funnel plot results are available from the authors on request). However, as shown in Table 3, the data did not indicate that there was a publication bias.

Thus, the Null Hypothesis 1 (H_0_1) is to be rejected, which proposed that there is no effect of EI on sports performance. The Alternative Hypothesis 1 (H_A_1) must be accepted, which proposed that there is an effect of EI and sports performance.

#### 3.2.2. Analysis of Moderators

Effect sizes in the domain conceptualisation of EI showed a minimum increased positive correlation for the studies that were assigned to the ability approach to EI and the studies assignment of the trait approach to EI (*r* = 0.19, *p* < 0.05). On the other hand, the effect size for the studies that assigned themselves to the mixed models of EI was not significant. The Q statistic did not indicate that the differences were significant, thus providing no grounds for rejecting the Null Hypothesis 5 (H_0_5).

The domain number of the citation demonstrated a slight increased correlation for studies which were cited between 11 and 50 times (*r* = 0.19, *p* < 0.01). Here, the Q statistic did not indicate that the differences were significant. Therefore, the Null Hypothesis 7 (H_0_7) was not rejected.

The results for the domain type of report demonstrated an increased significant effect size for non peer-reviewed documents (*r* = 0.20, *p* < 0.01), but the difference between them and the peer-reviewed documents (*r* = 0.14, *p* < 0.01) was not significant; hence, the Null Hypothesis 8 (H_0_8) was not rejected.

In the domain year of publication, for studies published between 2001 and 2009, effect size increased from small to moderate (*r* = 0.25, *p* > 0.05). For studies published since 2010, the effect size remained unchanged. The Q statistic did not provide that the differences were significant, and with that no reason to reject the Null Hypothesis 9 (H_0_9). Because of the limited number of available studies in the subgroups, we did not conduct moderator analyses for the following potential moderators: (a) type of sport (Research question 2); (b) level of expertise in sports (Research question 3); (c) measurement of sports performance (Research question 4); and (d) measurement of EI (Research question 6). Table 4 displays the main effect analyses of the categorical moderator variables.

## 4. Discussion

To our best knowledge, this is the first meta-analysis of the relationship between EI and sports performance in competitive sports. Compared to other more qualitative reviews, the advantage of conducting a meta-analysis is that it provides an estimate of the mean correlation of the strength of a relationship, given a whole population of studies. A total of 22 effect sizes were used for the quantitative analysis. The correlation between EI and sports performance demonstrated a small but significant association (*r* = 0.16).

Although small, the overall effect size suggested that higher EI has been linked to higher sports performance. For athletes in competitive sports a high EI could, hence, be beneficial. This finding supports the idea that EI is a weak determinant of sports performance and also confirms the conclusions of systematic reviews [5,30].

There are a number of possible reasons for why the higher scores in EI lead to a competitive advantage. Undoubtedly competitive sports is an emotion-laden environment [6,30,32,50]. The requirements for excellent performance in sports are diverse and challenging [90] and these demands for high performance are continually increasing [29].

First, athletes have to achieve long-term goals, planned far in advance and requiring many years of preparation. This implies ongoing motivation for intense and hard training as well as a continuous ability to cope with stress and pressure [91]. Secondly, adversities and emotional events (common mistakes, setbacks, and sustained injuries) must be overcome [3]. Finally, athletes need to perform precisely at the maximum. They have to decide promptly and under pressure how to react to environmental demands in order to achieve their performance goals [29,92].

This illustrates that athletes’ ability to perceive and express emotion, the ability to assimilate emotion in thought, understand and reason with emotion in the first place is an important prerequisite to recognise their performance states, to become conscious of the nature of their own emotions, and finally to define and implement the right decisions. Then the ability to regulate emotion to effectively cope with their emotions during sport participation in order to be able to control and influence their levels of energy is a relevant factor to control their level of energy in order to achieve optimal performance [1]. Regulation of emotion (e.g., effective stress management, frustration tolerance, mood regulation, and the exercise of emotional restraint, all the while within public purview and scrutiny) seems to be the tool with which the athlete creates and maintains positive affective states, which have been suggested to benefit sports performance [37]. 

Furthermore, competitive sports is fundamentally a social activity. Teamwork, team role responsibility, leadership, mutual goal-setting, problem-solving, cooperation, communication, and respect for others, etc. are challenges that are influenced by emotions [32,33,39,91].

Consequently, we can conclude that the ability to perceive, understand and reason emotions of teammates, coaches, referees, spectators and opponents in the first place is an essential prerequisite for professionalism in coping with emotional environmental demands. Then, athletes’ ability to regulate emotions of individuals emotions involved in sporting performance is necessary to control and influence social activity in order to be successful at the highest level of competition in the process of peak performance in competitive sport [93].

We were also interested in exploring potential moderators of the relation between EI and sports performance. We found that effect size did not depend significantly on the conceptualisation of EI. Taking this into account, we can assume that we did not find evidence for the superiority of a concept of EI.

This result is interesting, because research either assumed that each approach produce different results [23,94,95,96,97], or supported a preference for a specific EI concept [6]. One explanation might be the lack of theoretical discourse in many of the observed studies, meaning the allocation to one model of EI was not elaborated or discussed, or was not reflected in the corresponding measurement methods. For some studies an allocation was not even possible. The results of our meta-analysis do not support the assumption that conceptualisation of EI is a relevant determinant of the relationship between EI and sports performance. We did not find evidence for the superiority of an EI concept. However, further studies are needed to test this assumption explicitly.

We were also interested in carrying out quantitative analyses of other potential moderators such as “type of sport”, “level of expertise in sports”, “measurement of sports performance”, and “measurement of EI”. Unfortunately, the subgroups were too small (*k* < 5), so the estimate of effect size was imprecise and meaningful conclusions therefore inappropriate [98]. The questions of whether the effect size of the relation between EI and sports performance differs significantly with respect to these potential moderators could not be answered in the current investigation. More studies are required to give better indications.

Finally, we found that effect size of the relationship between EI and sports performance did not depend significantly on “publication types” and “number of citations”. Although peer-reviewed studies tended to report lower effect sizes, this difference was not significant. Furthermore, studies with the highest number of citations reported higher effect size, but this moderating effect was not significant either. We could not find any evidence that these forms of scientific publishing evaluations are relevant for the relationship between EI and sports performance. We noted the same for the potential moderator date of publication date. Although studies published between 2001 and 2009 tended to report higher effect sizes, this difference was not significant.

Almost all studies were cross-sectional. The strength of the relation between EI and sports performance was, therefore, determined by this study design. Furthermore, we found differences in the quality of the observed studies. As stated previously, seemingly basic demographic data was often omitted from studies and descriptions of the procedures used were often less than adequate. In some studies, important information was often missing or unclear.

## 5. Future Directions

Throughout the discussion we have identified recommendations for future research that have arisen from the current findings regarding our research questions and the overall review of the literature. First, future EI research should carefully consider selecting clear, objective, and comparable measures of sports performance across different sports and levels of sports performance. Second, future investigations should use a theoretically derived model of EI which is finally reflected in the corresponding operationalisation. Third, the great number of EI measurements and the resulting heterogeneity make it difficult to draw conclusions that will advance sport psychology research and practice. Therefore, it would be beneficial to find substantive consensus with regard to defining and measuring EI. Where appropriate, analysis providing a side-by-side comparison of ability and trait measures would be helpful. However, future investigations should first of all use measurements of EI, that are valid, reliable, and critically thought out with regard to their theoretical foundations and target outcomes. Good availability and practical handling of EI measurements would be desirable for future research work. Because of the large amount of heterogeneity in variables of sports performance, type of sport, and level of sports performance, we believe researchers should be encouraged to conduct and publish replication studies. Fifth, future research may want to examine the relationship between EI and types of performance measurement in greater detail.

Finally, the following general implications should also be noted here. First, the authors of future primary studies should use standardised guidelines for transparent reporting, which can subsequently be used in meta-analyses. Very basic statistics were often left out of results sections, making our calculations of effect sizes challenging and/or difficult to accomplish. Finally, a cumulative meta-analysis to complement and extend the current findings by including studies published after this review, as well as non-English, German, and Spanish ones (both before and after their study), is required to further explore the connection between EI and sports performance in competitive sports.

## 6. Limitations

There were a number of limitations to the current study. First, because of the limited number of study sizes, we were not able to examine all moderators, leaving several research questions we had raised unanswered. Second, the present quantitative analysis was dominated by studies using a cross-sectional design. Future studies should use longitudinal designs and conduct advanced analyses, such as multilevel analysis. Third, the current study relies on a linear model to determine the relationship between EI and sports performance. It is possible that such a relationship depends on multiple factors in a non-linear fashion and that this changes over time. In that case, the linear assessment of data collected over a number of years might have contributed to the low effect sizes in the current meta-analysis.

Fourth, we reported considerable variability in measurements used to assess EI. The convergent validity of different EI measurements, both within and between EI models, has long been recognised as a concern in the EI literature [15,28,99,100]. Meta-analysis methodology often includes combining different operationalisation of the same variable [64]. However, the general lack of convergence among EI measurements suggests a cautious interpretation of our findings. The fifth limitation was the quality of the included studies. We did not summarise scores, because this involves inherent weighting of component items, some of which may not be directly related to the validity of a study’s findings. It would also not be clear how weights for different items should be determined, and different scales might have reached different conclusions on the overall quality of an individual study [101]. However, we did not control the quality, as primary sources on meta-analysis recommend against this practice [64]. Sixth, including only published sources written in English, German, or Spanish could potentially lead to a publication bias in a meta-analysis. However, tests we conducted indicated there was no evidence of publication bias. The last limitation of our findings is the heterogeneity of the studies.

Regarding this, Higgins [102] noted, that “heterogeneity is to be expected in a meta-analysis: it would be surprising if multiple studies, performed by different teams in different places with different methods, all ended up estimating the same underlying parameter”(p. 1158).

Finally, it is important to be aware that the methodologies of the meta-analysis and the studies on which it is based do not provide evidence regarding causality.

## 7. Conclusions

The results of this meta-analysis suggest that EI is a weak determinant of sports performance. The quantitative analyses demonstrated a small positive relation between EI and sports performance. We did not find evidence that the conceptualisation of EI moderates this relationship. The same applies to the publication type, number of citations, and publication date. Overall, the results seem encouraging regarding the value of EI as a possible sports performance predictor. Practitioners, such as applied sport psychologists, coaching staff, athletes, and sport administrators, therefore, need appropriate knowledge of the role of EI and its relevance for successful performance in major competitions. Based on such facts, practitioners should promote the implementation of EI screening and EI-development programs as an integral part of the training process. This, however, requires a more intensive exploration and supply of useable conceptualisations of EI in sports as well as valid and easily handled instruments of EI and, finally, well-grounded and easily implemented EI-training programs. 

## Figures and Tables

**Table 1:** Summary of all studies examining emotional intelligence (EI) and sports performance in competitive sports.

Authors (Year)	Setting	Design	N	Age	Type of Sport	Performance Level	Type of EI Concepts	EI Measurement—Authors (Year)—Items	Measurement of Sports Performance
[78] Araya, G.A., and Salazar, W. (2001)	CR	Cross-sectional	10	*Range* = 17–25	Team sport	From amateurs to elite athletes	A clear allocation not possible	Prueba de inteligencia emocional —Goleman (1997) —Not specified	Statistical accounts —Basketball precision shooting tests
[79] Arribas-Galarraga, S., Saies, E., Cecchini, J.A., Arruza, J.A., and Luis-De-Cos, I. (2017)	ESP	Cross-sectional	386	Not specified	Individual sport	From amateurs to elite athletes	A clear allocation not possible	Un Modelo de medida de la Inteligencia Emocional percibida en contextos deportivo/competitivos) —Arruza et al. (2013) —39 Items	Statistical accounts —Performance Index
[80] Bal, B.S. and Singh, D. (2014)	IND	Cross-sectional	60	*Range* = 19–25	Team sport	From amateurs to elite athletes	A clear allocation not possible	Emotional Intelligence Scale (EIS) —Hyde, Pethe, and Dhar (2002) —Not specified	Level of expertise —League-membership
[77] Berry, D.O. (2013)	USA	Cross-sectional	101	*Range* = 18–27	Team sport	Professional athletes	Ability approach	The Mayer-Salovey-Caruso Emotional Intelligence Test (MSCEIT)—(Expert-Consense Method) —Mayer, Salovey, and Caruso, 2002 —141 Items	Statistical accounts —Team performance
[37] Crombie, D., Lombard, C. and Noakes, T. (2009)	ZAF	Longitudinal	121	Not specified	Teamsport	Professional athletes	Ability approach	The Mayer-Salovey-Caruso Emotional Intelligence Test (MSCEIT)—(Expert-Consense Method) —Mayer, Salovey, and Caruso, 2002 —Not specified	Statistical accounts —Game statistics
[81] Dimick, J.A. (2017)	USA	Cross-sectional	159	*Range* = 18–24	Individual and team sport	Professional athletes	Mixed models	Emotional Intelligence Appraisel (2015) —TalentSmart (2010) —28 Items	Level of expertise —Starter/ non-starter
[82] Hemmatinezhad, M.A., Ramazaninezhad, R., Ghezelsefloo, H. and Hemmatinezhad, M. (2012)	IRN	Cross-sectional	95	*M* = 21.5 *SD* = 2.3	Team sport	Professional athletes	A clear allocation not possible	Emotional Intelligence Scale (EIS) —Not specified —Not specified	Statistical accounts —Game statistics
[83] Karimi Khiavi, Z. and Taghi Aghdasi, M. (2014)	IRN	Cross-sectional	376	A clear allocation not possible	Individual and team sport	From amateurs to elite athletes	A clear allocation not possible	Vyzynger Emotional Intelligence Questionnaire —Not specified —25 Items	Assessment of sports performance —Defense styles questionnaire (DSQ) —Questionnaire Athletic Performance
[38] Laborde, S., Dosseville, F., Guillén, F. and Chávez, E. Study 1 (2014)	FRA/GER	Cross-sectional	973	*M* = 21.4 *SD* = 3.9 *Range* = 17–56	Individual and team sport	From amateurs to elite athletes	Trait approach	The Trait Emotional Intelligence Questionnaire—French version (TEIQue) —Petrides, K.V. (2009) —153 items	Level of expertise —Assessed by self-report
[38] Laborde, S., Dosseville, F., Guillén, F. and Chávez, E. Study 2 (2014)	FRA/GER	Cross-sectional	291	Ecuador-Athletes (128) *M* = 22.4 *SD* = 4.4 *Range* = 17–40 Spain-athletes (163) *M* = 22.7 *SD* = 4.7 *Range* = 18–39	Not specified	Not specified	Trait approach	The Trait Emotional Intelligence Questionnaire—spanish version (TEIQue) —Petrides, K.V. (2009) —153 items	Assessment of sports performance —Performance Satisfaction based on Nicholls, Polman, and Levy (2010).
[42] Laborde, S., Lautenbach, F., Allen, M.S., Herbert, C., Achtzehn, S. (2014)	FRA/GER	Quasi-experimental	28	*M* = 23.9 *SD* = Not specified *Range* = 16–36	Individual sport	Amateur athletes	Trait approach	The Trait Emotional Intelligence Questionnaire—german version (TEIQue) —Freudenthaler, Neubauer, Gabler, Scherl, and Rindermann, 2008 —153 items	Statistical accounts —Tennis serving task
[84] Martínez Ferreiro, J. (2016)	ESP	Cross-sectional	30	*Range* = 18–33	Team sport	Professional athletes	Trait approach	Trait Meta-Mood Scale (TMMS) (Salovey et al., 1995)—spanish version —Fernández-Berrocal et al. (2004) —24 Items	Statistical accounts —Playing minutes
[85] Mitić, P., Mitrović, M., Bratić, M. and Nurkić, M. (2011)	SRB	Cross-sectional	44	*Range* = 17–24	Individual sport	From amateurs to elite athletes	A clear allocation not possible	Emotional Competence Questionnaire—UEK-45 —Takšić, V., Moharić, T. and Munjas, R. (2006) —45 Items	Assessment of sports performance —Assessed by coaches
[86] Mohammad, G., Khan, S., and Singh, J. (2015)	IND	Cross-sectional	200	*Range* = 16–27	Team sport	Professional athletes	A clear allocation not possible	Mangal emotional intelligence inventory —Mangal and Mangal (2004) —100 Items	Level of expertise —League-membership
[39] Perlini, A. and Halverson, T. (2006)	CAN	Cross-sectional	79	*M* = 28.8 *SD* = 3.8 *Range* = 23–37	Team sport	Professional athletes	Mixed models	The Emotional Quotient Inventory (Bar’On EQ-i) —Bar-On 1997 —133 Items	Statistical accounts —Game statistics
[40] Petrides, K.V., Niven, L. and Mouskounti, T. (2006)	GBR	Cross-sectional	34	*M* = 18 *SD* = 0.7	Individual sport	Elite athletes	Trait approach	Trait Emotional Intelligence Questionnaire (TEIQue) —Petrides (2009) —153 Items	Assessment of sports performance —Assessed by coaches
[80] Saies, E., Arribas-Galarrag, S., Cecchini, J.A., Luis-De-Cos, I. and Otaegi, O. (2014)	ESP	Cross-sectional	347	Not specified	Individual sport	Professional athletes	Ability approach	Un Modelo de medida de la Inteligencia Emocional percibida en contextos deportivo/competitivos) —Arruza et al. (2013) —39 Items	Level of expertise —Cuestionario de Satisfacción con los Resultados Deportivos (CSRD)
[87] Soflu, H.G., Esfahani, N. and Assadic, H. (2011)	IRN	Cross-sectional	160	Not specified	Individual and team sport	Professional athletes	Mixed models	Emotional intelligence questionnaire —Not specified —Not specified	Level of expertise —Not specified
[88] Stough, C., Clements, M., Wallish, L. and Downey, L. (2009)	AUS	Cross-sectional	49	Female: *M* = 18.1 *SD* = 2.3 *Range* = 15–27 Male:*M* = 18.3 *SD* = 6.3 *Range* = 11–35	Individual sport	Professional athletes	Mixed models	Swinburne University Emotional Intelligence Test (SUEIT) —Palmer and Stough (1999) —77 Items	Statistical accounts —Game statistics
[41] Tok, S., Binboğa, E., Guven, S., Çatıkkas, F., and Dane, S. (2013)	TR	Cross-sectional	52	Not specified	Individual and team sport	Not specified	A clear allocation not possible	Measure of emotional intelligence —Schutte et al. (1998) —revised by Austin (2004) —adapted by Tok et al. (2005) —41 Items	Physiological Parameters —Maximum voluntary contraction (MVC)
[34] Zizzi, S.J. Deaner, H. and Hirschhorn, D.K. (2003)	USA	Cross-sectional	61	*Range* = 18–23	Individual sport	Professional athletes	Ability approach	Measure of Emotional Intelligence —Schutte et al. (1998) —33 Items	Statistical accounts —Game statistics

AUS = Australia; CAN = Canada; CR = Costa Rica; ESP = Spain; FRA = France; GER = Germany; GBR = United Kingdom; IND = India; IRN = Iran; SRB = Republic of Serbia; TR = Turkey; USA = United States; ZAF = South Africa; *M* = mean; *SD* = standard deviation; *N* = participants; The sample numbers present the absolute sample size of the studies.

**Table 2:** Quality assessment–individual evaluation of the studies examined.

Authors (Year)	Citations	PR	Items Strengthening the Reporting of Observational Studies in Epidemiology (STROBE) Statement														
			1	2	3	4	5	6	7	8	9	10	11	12	13	14	15
[78] Araya et al. (2001)	6	No	Yes	No	Partly	Partly	No	Yes	No	No	No	No	No	Partly	No	No	No
[79] Arribas-Galarraga et al. (2017)	2	No	Yes	No	No	No	Yes	Yes	No	No	No	Yes	No	Partly	No	No	No
[89] Bal et al. (2014)	0	No	Partly	No	Partly	Partly	No	Partly	No	No	No	Yes	No	No	Yes	No	No
[77] Berry (2013)	1	Yes	Yes	Yes	Yes	Yes	Yes	Yes	No	Yes	Yes	Yes	Yes	Yes	Yes	No	Yes
[37] Crombie et al. (2009)	85	Yes	Yes	Yes	Yes	No	Yes	Yes	No	No	Yes	Yes	No	Partly	Yes	Yes	Yes
[81] Dimick (2017)	0	Yes	Yes	No	Yes	No	No	Yes	No	No	No	Yes	No	Partly	Yes	No	Yes
[82] Hemmatinezhad et al. (2012)	5	Yes	No	No	No	Yes	No	Yes	No	No	No	Partly	No	No	Yes	No	No
[83] Karimi Khiavi et al. (2014)	0	No	Partly	Yes	No	No	No	Yes	No	No	No	No	No	Yes	Yes	No	No
[42] Laborde et al. (2014)	71	Yes	Yes	Yes	Yes	Yes	Partly	Yes	Yes	No	Yes	Yes	No	Partly	Yes	Yes	Yes
[38] Laborde et al. (2014)																	
Sample 1	43	Yes	Yes	Yes	Yes	Yes	Yes	Yes	No	No	Yes	Yes	No	Yes	Yes	Yes	No
Sample 2	43	Yes	Yes	Yes	Yes	Yes	No	Yes	No	No	Yes	Yes	No	Yes	Yes	Yes	No
[84] Martínez Ferreiro (2016)	0	Yes	Yes	Yes	Yes	Partly	No	Yes	No	No	Yes	Yes	No	Yes	Yes	Yes	Yes
[85] Mitić et al. (2011)	4	Yes	Yes	No	Yes	No	No	Yes	No	No	Yes	No	No	No	Yes	Yes	Yes
[86] Mohammad et al. (2015)	?	No	No	No	No	No	No	Partly	No	No	No	Yes	No	No	Partly	No	No
[39] Perlini et al. (2006)	111	Yes	Yes	Yes	Yes	Yes	Yes	Yes	No	No	Yes	No	No	Yes	Yes	Yes	Yes
[40] Petrides et al. (2006)	82	Yes	Yes	No	No	Yes	No	Yes	No	No	No	No	No	No	Yes	No	Partly
[80] Saies et al. (2014)	15	Yes	Yes	Yes	No	Yes	Yes	Yes	No	No	Yes	Yes	No	No	Yes	Yes	Yes
[87] Soflu et al. (2011)	9	Yes	Yes	No	No	Yes	No	Yes	No	No	No	Yes	No	No	Yes	No	No
[88] Stough et al. (2009)	23	No	Yes	Yes	No	No	Yes	No	No	No	No	No	No	No	Yes	No	No
[41] Tok et al. (2013)	12	Yes	Yes	Yes	Yes	Partly	No	Yes	No	No	No	Yes	No	No	No	Yes	No
[34] Zizzi et al. (2003)	159	Yes	Yes	No	Yes	Yes	Yes	Yes	No	No	No	No	No	Partly	Yes	Yes	Yes

Search for citations was conducted on 2 August 2018 with Google Scholar. PR = Peer-reviewed; 1 = State specific objectives; 2 = State pre-specified hypotheses; 3 = Present key elements of study design early in the paper; 4 = Describe the setting; locations, and relevant dates; 5 = Give the eligibility criteria, and the sources and methods of selection of participants; 6 = Describe all variables and how there were measured; 7 = Describe any efforts to address potential sources of bias; 8 = Explain how the study size was arrived at; 9 = Explain how quantitative variables were handled in the analyses; 10 = Describe all statistical methods, including those used to control for confounding; 11 = Explain how missing data points were addressed; 12 = Give characteristics of study participants (e.g., demographic, clinical, social); 13 = Report numbers of outcome events or summary measures; 14 = Discuss limitations of the study, taking into account sources of potential bias or imprecision; 15 = Discuss the generalisability (external validity) of the study result; Yes = Available; Partly = Partly available; No = Not available.

**Table 3:** Meta-analysis results for emotional intelligence and sports performance.

		Statistic for Each Study					Publication bias		
First Author (Year)	N	R	95% CI	^%^ weight	Study Residual	Study Removed	Fail-Safe N	(a)	r_ADJUSTED_
[78] Araya et al. (2001)	10	0.62 *	[−0.01, 0.90]	0.61	1.47	0.16			
[79] Arribas-Galarraga et al. (2017)	386	0.16 **	[0.07, 0.26]	8.86	0.01	0.16			
[89] Bal et al. (2014)	60	0.15	[−0.11, 0.39]	3.62	−0.12	0.16			
[77] Berry (2013)	97	0.05	[−0.16, 0.24]	4.98	.0.90	0.17			
[37] Crombie et al. (2009)	12	0.69 **	[0.19, 0.91]	0.77	1.99	0.16			
[81] Dimick (2017)	69	−0.01	[−0.25, 0.22]	4.00	−1.20	0.17			
[82] Hemmatinezhad et al. (2012)	95	0.21 *	[0.01, 0.40]	4.92	0.37	0.16			
[83] Karimi Khiavi et al. (2014)	376	0.21 **	[0.11, 0.30]	8.80	0.46	0.16			
[42] Laborde et al. (2014)	28	0.25	[−0.14, 0.57]	1.91	0.42	0.16			
[38] Laborde et al. (2014)									
Sample 1	973	0.05	[−0.01, 0.11]	10.46	−1.29	0.18			
Sample 2	291	0.25 **	[0.14, 0.36]	8.18	0.90	0.16			
[84] Martínez Ferreiro (2016)	30	0.20	[−0.18, 0.52]	2.04	0.16	0.16			
[85] Mitić et al. (2011)	22	0.27	[−0.17, 0.62]	1.51	0.47	0.16			
[86] Mohammad et al. (2015)	200	0.25 **	[0.12, 0.38]	7.15	0.87	0.16			
[39] Perlini et al. (2006)	79	−0.16	[−0.37, 0.06]	4.38	−2.31	0.18			
[40] Petrides et al. (2006)	34	0.35 *	[0.01, 0.62]	2.29	1.02	0.16			
[80] Saies et al. (2014)	347	0.23 **	[0.13, 0.33]	8.61	0.69	0.16			
[87] Soflu et al. (2011)	160	0.03	[−0.13, 0.18]	6.49	−1.21	0.17			
[88] Stough et al. (2009)	49	0.25	[−0.03, 0.50]	3.10	0.55	0.16			
[41] Tok et al. (2013).	52	0.26	[−0.01, 0.50]	3.25	0.62	0.16			
[34] Zizzi et al. (2003)									
Sample 1	21	0.34	[−0.11, 0.67]	1.44	0.76	0.16			
Sample 2	40	0.01	[−0.30, 0.32]	2.63	−0.83	0.17			
**Overall** (*k* = 22) *Random effect model*	**3431**	0.16 **	**[0.11, 0.22]**				**369**	**0.85**	0.15 **

*N* = sample size; *r* = effect size (Pearson’s *r*); *CI* = confidence interval [lower limit, upper limit]; ^%^ weight = relative weight; study removed = shows the overall mean weighted effect size *r* for all studies, except the study in each row; Fail-safe *N* = number of studies needed to nullify a significant effect; (a) = intercept value Egger test; r_ADJUSTED_= adjusted estimate of effect (if publication bias is present) through an iterative statistical procedure to calculate a symmetrical funnel plot; * *p* < 0.05; ** *p* < 0.01.

**Table 4:** Outcomes of the exploratory analysis of the correlation between emotional intelligence and sports performance.

	Sample		Effect Size Statistic			Heterogeneity	
Subgroups	*K*	*N*	*r*	95% *CI*	*Z*	*Q*	*p*
**Type of sport**							
Team sport	13	1109	0.16 **	[0.07, 0.25]	3.28	0.29	0.96
Individual sport	4	470	0.19 **	[0.10, 0.27]	4.04		
Both of them	3	588	0.1 5*	[0.02, 0.29]	2.21		
A clear allocation not possible	2	1264	0.15	[−0.05, 0.33]	1.43		
**Level of expertise in sports**							
Elite athletes	2	56	0.32 *	[0.06, 0.54]	2.35	4.52	0.34
Professional athletes	12	1199	0.14 **	[0.04, 0.23]	2.80		
Amateur athletes	1	28	0.25	[−0.14, 0.57]	1.28		
From elite to amateur athletes	5	1805	0.15 **	[0.05, 0.24]	2.94		
A clear allocation not possible	2	343	0.25 **	[0.15, 0.35]	4.72		
**Measurement of sports performance**							
Level of expertise	6	1809	0.12 *	[0.03, 0.22]	2.50	3.89	0.27
Statistical accounts	11	847	0.16 **	[0.05, 0.26]	2.75		
Assessment of sports performance	4	723	0.23 **	[0.16, 0.30]	6.31		
Physiological parameters	1	52	0.26	[−0.01, 0.50]	1.86		
**Conceptualisation of EI**							
Ability approach to emotional intelligence	5	517	0.19 *	[0.02, 0.35]	2.20	4.98	0.17
Mixed models of emotional intelligence	5	379	0.03	[−0.11, 0.17]	0.48		
Trait approach to emotional intelligence	5	1356	0.19 *	[0.04, 0.32]	2.53		
A clear allocation not possible	7	1179	0.20 **	[0.15, 0.26]	7.01		
**Measurement of EI**							
self-report	20	3322	0.16 **	[0.11, 0.22]	5.47	0.30	0.58
ability-test	2	109	0.37	[−0.37, 0.82]	0.97		
**Number of citations**							
0–10	10	1305	0.15 **	[0.09, 0.21]	4.88	2.31	0.51
11–50	5	1712	0.19 **	[0.07, 0.30]	3.18		
>50	6	214	0.21	[−0.05, 0.43]	1.59		
Not specified	1	200	0.25 **	[0.12, 0.38]	3.64		
**Peer reviewed document**							
Peer-reviewed documents	16	2350	0.14 **	[0.06, 0.22]	3.54	1.50	0.22
Non peer-reviewed documents	6	1081	0.20 **	[0.14, 0.26]	6.68		
**Year of publication**							
2001–2009	7	244	0.25 *	[0.01, 0.46]	2.00	0.47	0.49
2010–2018	15	3187	0.16 **	[0.11, 0.22]	5.77		

Random effect model. *k* = study numbers; *N* = total sample size; *r* = effect size (Pearson’s *r*); *CI* = confidence interval [lower limit, upper limit]; *Z* = *Z*-Value; * *p* < 0.05; ** *p* < 0.01; *Q* = *Q*-value total between subgroups. *p* = *p*-value.

## Supplementary Materials

The following are available online at http://www.mdpi.com/2075-4663/6/4/175/s1, Table S1: PRISMA 2009 checklist.
