# Supplementary material for: The Influence of Emotional Intelligence on Performance in Competitive Sports: A Meta-Analytical Investigation
Source: Sports (Basel). 2018 Dec 13;6(4):175. doi: 10.3390/sports6040175 (PMC6316207; doi:10.3390/sports6040175)
Supplement: Supplementary file 1 [file sports-06-00175-s001.pdf]

PRISMA 2009 Checklist

Section/topic	#	Checklist item	Reported on page #
**TITLE**			
Title	1	Identify the report as a systematic review, meta-analysis, or both.	1
**ABSTRACT**			
Structured summary	2	Provide a structured summary including, as applicable: background; objectives; data sources; study eligibility criteria, participants, and interventions; study appraisal and synthesis methods; results; limitations; conclusions and implications of key findings; systematic review registration number.	1
**INTRODUCTION**			
Rationale	3	Describe the rationale for the review in the context of what is already known.	1-5
Objectives	4	Provide an explicit statement of questions being addressed	3-5
**METHODS**			
Protocol and registration	5	Indicate if a review protocol exists, if and where it can be accessed (e.g., Web address), and, if available, provide registration information including registration number.	6
Eligibility criteria	6	Specify study characteristics (e.g., PICOS, length of follow-up) and report characteristics (e.g., years considered, language, publication status) used as criteria for eligibility, giving rationale.	5-6
Information sources	7	Describe all information sources (e.g., databases with dates of coverage, contact with study authors to identify additional studies) in the search and date last searched.	6, Protocol
Search	8	Present full electronic search strategy for at least one database, including any limits used, such that it could be repeated.	6, Protocol
Study selection	9	State the process for selecting studies (i.e., screening, eligibility, included in systematic review, and, if applicable, included in the meta-analysis).	6 (Fig. 1.)
Data collection process	10	Describe method of data extraction from reports (e.g., piloted forms, independently, in duplicate) and any processes for obtaining and confirming data from investigators.	7-8
Data items	11	List and define all variables for which data were sought (e.g., PICOS, funding sources) and any assumptions and simplifications made.	8
Risk of bias in individual studies	12	Describe methods used for assessing risk of bias of individual studies (including specification of whether this was done at the study or outcome level)	8
Summary measures	13	State the principal summary measures (e.g., risk ratio, difference in means).	8-9
Synthesis of results	14	Describe the methods of handling data and combining results of studies, if done, including measures of consistency (e.g., I^2) for each meta-analysis.	8-9

PRISMA 2009 Checklist

Section/topic	#	Checklist item	Reported on page #
Risk of bias across studies	15	Specify any assessment of risk of bias that may affect the cumulative evidence (e.g., publication bias, selective reporting within studies).	9
Additional analyses	16	Describe methods of additional analyses (e.g., sensitivity or subgroup analyses, meta-regression), if done, indicating which were pre-specified.	9
**RESULTS**			
Study selection	17	Give numbers of studies screened, assessed for eligibility, and included in the review, with reasons for exclusions at each stage, ideally with a flow diagram.	9-10; Figure 1.
Study characteristics	18	For each study, present characteristics for which data were extracted (e.g., study size, PICOS, follow-up period) and provide the citations.	9; Table 1.
Risk of bias within studies	19	Present data on risk of bias of each study and, if available, any outcome level assessment (see item 12).	19; Table 2.
Results of individual studies	20	For all outcomes considered (benefits or harms), present, for each study: (a) simple summary data for each intervention group (b) effect estimates and confidence intervals, ideally with a forest plot.	Table 3.
Synthesis of results	21	Present results of each meta-analysis done, including confidence intervals and measures of consistency.	22; Table 3.
Risk of bias across studies	22	Present results of any assessment of risk of bias across studies (see Item 15).	22; Table 3.
Additional analysis	23	Give results of additional analyses, if done (e.g., sensitivity or subgroup analyses, meta-regression [see Item 16]).	25; Table 4.
**DISCUSSION**			
Summary of evidence	24	Summarize the main findings including the strength of evidence for each main outcome; consider their relevance to key groups (e.g., healthcare providers, users, and policy makers).	28-29
Limitations	25	Discuss limitations at study and outcome level (e.g., risk of bias), and at review-level (e.g., incomplete retrieval of identified research, reporting bias).	29-30
Conclusions	26	Provide a general interpretation of the results in the context of other evidence, and implications for future research.	28-29
**FUNDING**			
Funding	27	Describe sources of funding for the systematic review and other support (e.g., supply of data); role of funders for the systematic review.	30

From: Moher D, Liberati A, Tetzlaff J, Altman DG, The PRISMA Group (2009). Preferred Reporting Items for Systematic Reviews and Meta-Analyses: The PRISMA Statement. PLoS Med 6(7): e1000097. doi:10.1371/journal.pmed1000097

Section and topic			Item	Checklist item	
**ADMINISTRATIVE INFORMATION**					
Titel			1.	The influence of emotional intelligence on performance in competitive sports: A meta-analytical investigation	
Registration			2.	No registration	
Authors:			3.	Alexandra Kopp<br>Alexandra.kopp@hu-berlin.de<br>Institutional Address: Humboldt-Universität zu Berlin, Kultur-, Sozial- und Bildungswissenschaftliche Fakultät, Institut für Sportwissenschaft, Philippstraße 13, 10115 Berlin<br>Postal Address: Humboldt-Universität zu Berlin, Kultur-, Sozial- und Bildungswissenschaftliche Fakultät, Institut für Sportwissenschaft, Unter den Linden 6, 10099 Berlin<br><br>Darko Jekauc	
Support:			5.	No supporting.	
**INTRODUCTION**					
Rationale			6.	The reason for conducting this meta-analysis was to clarify the literature available. No prior meta-analyses have examined EI and sports performance, so the following relationships have not been tested using meta-analytical techniques. We will address this by using meta-analysis to more accurately determine the overall size of the relationships between EI and sports performance. In addition to this main effect, we will examine several potential conceptual and empirical moderators of the relationship between EI and sports performance.	
Objectives			7.	Research Question 1: Is emotional intelligence related to sports performance in competitive sports?<br>Research Question 2: Is there a difference between effect size between individual sports athletes and team sports athletes?<br>Research Question 3: Does effect size depend on sports expertise?<br>Research Question 4: Does effect size depend on measurements of sports performance?<br>Research Question 5: Does effect size depend on the concept of EI?<br>Research Question 6: Is there a difference in effect size between assessment of EI via self-report and assessment of EI via ability-test?	
**METHODS**					
Eligibility criteria			8.	(1) The study was empirical in nature.<br>(2) The study involved quantitative methods.<br>(3) One independent variable was EI.<br>(4) The dependent variable was sports performance.<br>(5) The sample was constituted of sport competition participations.<br>(6) The article was written in English, German or Spanish.	
Search strategy:	Electronic database		9 to 10	SPORTDiscus, PsycARTICLES, PsycINFO, PubMed, and Scopus	online - Search: 28 June 2018
	Keywords			(emotional intelligence or emotional competence) and (sport or athletics or athletes or competitive sport or sports performance)	Title - Screening: 28 June 2018
	Searchfilters	Publication		All	Abstract - Screening: 2rd July
		Setting		All	Fulltext - Screening: 3rd July
		Type of		All	Fulltext additional literature:
		Language		English, German or Spanish	Flowdiagram: 4rd July 2018
	Contact with study authors			Laborde 4rd July 2018 - ongoing studies?<br>Ros 30 June 2018 literature request?	
	Data management		11a	Protokoll for search history<br>PRISMA-Checklist	
	Selection process		11b	Two independent reviewers through each following phase of the review: screening, eligibility and inclusion in meta-analysis.	Dataextraction: 5rd July - 9. July 2018
	Data collection process		11c	All studies were coded independently by two of the authors, and their ratings were compared. Any disagreements were resolved by discussion and consensus.	Coding: 16rd July 2018

Study records:	Data items	11d	(1) Authors (year)_number (2) Study_ID_2015 (3) Year (4) Setting (5) Type of report (6) AV_measurement (7) Design (8) N (9) Performance level (10) Sport (11) Female percent (12) Age average (13) EI measurement (14) Type of EI –Inventory (15) Type of EI concepts (16) UV (17) Results (18) Effect size (Pearson's r) (19) peer-reviewed document?<br><br><u>Quality Assessment</u><br><br>(1) Peer-reviewed document? (2) State specific objectives. (3) State prespecified hypotheses. (4) Present key elements of study design early in the paper. (5) Describe the setting, locations, and relevant dates. (6) Give the eligibility criteria, and the sources and methods of selection of participants. (7) Describe all variables and how there were measured. (8) Describe any efforts to address potential sources of bias. (9) Explain how the study size was arrived. (10) Explain how quantitative variables were handled in the analyses. (11) Describe all statistical methods, including those used to control for confounding. (12) Explain how missing data were addressed. (13) Give characteristics of study participants (eg demographic, clinical, social). (14) Report numbers of outcome events or summary measures. (15) Discuss limitations of the study, taking into account sources of potential bias or imprecision. (16) Discuss the generalizability (external validity) of the study results.	The number of citations in Google Scholar was counted on 3rd August 2018
	Coding	12d	(4) Setting: AUS = Australia; CAN = Canada; CR = Costa Rica; ESP = Spain; FRA = France; GER = Germany; GBR = United Kingdom; HRV = Croatia; HUN = Hungary; IND = India; IRN = Iran; QAT = Qatar; SRB = Republic of Serbia; TR = Turkey; TWN = Taiwan; USA = United States; ZAF = South Africa;<br>(5) Type of report: 1 = article in a journal 2 = article in an book 3 = other<br>(6) AV_measurement: 1 = Level of expertise/ League-membership 2 = Statistical accounts for sport 3 = Assessment of sports performance 4 = physiological parameters<br>(7) Design: 1 = Experimental 2 = Cross-section 3 = Longitudinal section 4 = Quasi-experimental<br>(9) Performance level: 1 = Elite athletes 2 = Professional athletes 3 = Amateur athletes 4 = From amateurs to elite athletes 999 = Not specified<br>(14) Type of EI –Inventory: 1 = Self report 2 = Ability test<br>(15) Type of EI concepts: 1 = Ability approach to emotional intelligence 2 = Mixed models of emotional intelligence 3 = Trait approach to emotional intelligence 999 = A clear allocation not possible<br>(16) UV: 1 Self - Awareness 2 Self – Management 3 Social – Awareness 4 Social – Management 5 Using Emotion 6 EI – total - Score<br>(19) Peer-reviewed document?: 0 = No 1 = Peer reviewed 3 = Can't tell<br><br>Qualitative Auswertung:<br>1 = Yes; 2 = No; 3 = Partly	
Data synthesis		13.	Correlation (r) was used as the effect size because the majority of the research reported r, as it is easily calculated from chi-square, t, f, and d values, and it is readily understood and interpreted [49]. For studies based on group differences that did not report the effect size, but had given enough details to calculate an effect size, we first calculated the standardised mean differences (Cohen's d) and computed this effect size to r. If the same EI measurement was correlated with multiple performance measurements, provided they belonged to the same type of performance measurement, we averaged the correlations to obtain the effect size [48].<br>Comprehensive Meta-Analysis software (CMA), Version 2.0, was used. A random effects model was used to interpret findings [48,50]. The I-square (I2) value provides an estimate of the overlap of confidence intervals and is interpreted as low (25%), moderate (50%), or high (75%) values of the total variance attributed to covariates [54]. If I2 is high, then heterogeneity is very high, and the use of the random effect model for meta-analysis is justified [55]. Decisions made concerning retention or exclusion of outliers, i.e. large residual values two standard deviations (±1.96) above or below the study's mean effect size, were based on whether overall results remained significant ($p \leq .05$) and within the 95% confidence interval. The influence of individual studies on the overall mean weighted r was investigated by removing one study at a time from the overall analysis (one-study removed analysis) by using a 'one study removed' procedure in CMA.	Data synthesis: 1st August 2018
Meta-bias(es)		14.	Methods used to identify and control for publication bias included review of the funnel plot, the Egger's test for detecting asymmetry in a funnel plot [58], a Fail-safe N calculation [56], and a 'trim and fill' procedure [59]. Each study included the number of participants, the effect size (Pearson's r), the confidence interval (lower limit, upper limit), the relative weight, the residual value and the summary effect size if the study removed it.	Publicationbias & moderator analyses: 13rd August 2018
Moderator-analysis		15.	We will use Q statistic to test differences across moderators. A statistically significant Q statistic suggests that the heterogeneity exists in effect size distribution (i.e., the potential existence of moderators).	
